# Digital Game Interventions for Youth Mental Health Services (Gaming My Way to Recovery): Protocol for a Scoping Review

**DOI:** 10.2196/13834

**Published:** 2020-06-24

**Authors:** Manuela Ferrari, Sarah V McIlwaine, Jennifer Ann Reynolds, Suzanne Archie, Katherine Boydell, Shalini Lal, Jai L Shah, Joanna Henderson, Mario Alvarez-Jimenez, Neil Andersson, Jill Boruff, Rune Kristian Lundedal Nielsen, Srividya N Iyer

**Affiliations:** 1 Douglas Mental Health University Institute Montreal, QC Canada; 2 Department of Psychiatry McGill University Montreal, QC Canada; 3 Research Chair on Gambling Concordia University Montreal, QC Canada; 4 Department of Psychiatry and Behavioural Neurosciences McMaster University Hamilton, ON Canada; 5 Black Dog Institute Sydney Australia; 6 School of Rehabilitation Faculty of Medicine, University of Montreal Montreal, QC Canada; 7 Health Innovation and Evaluation Hub University of Montreal Hospital Research Centre Montreal, QC Canada; 8 Centre for Addiction and Mental Health Toronto, ON Canada; 9 Orygen University of Melbourne Melbourne Australia; 10 Department of Family Medicine McGill University Montreal, QC Canada; 11 Department of Pharmacology and Therapeutics McGill University Montreal, QC Canada; 12 Centre for Computer Games Research IT University of Copenhagen Copenhagen Denmark

**Keywords:** mental health, mental disorders, biomedical technology, video games, virtual reality, mental health services

## Abstract

**Background:**

Digital or video games are played by millions of adolescents and young adults around the world and are one of the technologies used by youths to access mental health services. Youths with mental health problems strongly endorse the use of technologies, including mobile and online platforms, to receive information, support their treatment journeys (eg, decision-making tools), and facilitate recovery. A growing body of literature explores the advantages of playing digital games for improving attention span and memory, managing emotions, promoting behavior change, and supporting treatment for mental illness (eg, anxiety, depression, or posttraumatic stress disorder). The research field has also focused on the negative impact of video games, describing potential harms related to aggression, addiction, and depression. To promote clarity on this matter, there is a great need for knowledge synthesis offering recommendations on how video games can be safely and effectively adopted and integrated into youth mental health services.

**Objective:**

The Gaming My Way to Recovery scoping review project assesses existing evidence on the use of digital game interventions within the context of mental health services for youths (aged 11-29 years) using the stepped care model as the conceptual framework. The research question is as follows: For which youth mental health conditions have digital games been used and what broad objectives (eg, prevention, treatment) have they addressed?

**Methods:**

Using the methodology proposed by Arksey and O’Malley, this scoping review will map the available evidence on the use of digital games for youths between 11 and 29 years old with mental health or substance use problems, or both.

**Results:**

The review will bring together evidence-based knowledge to assist mental health providers and policymakers in evaluating the potential benefits and risks of these interventions. Following funding of the project in September 2018, we completed the search in November 2018, and carried out data screening and stakeholder engagement activities during preparation of the protocol. We will conduct a knowledge synthesis based on specific disorders, treatment level and modality, type of service, population, settings, ethical practices, and user engagement and offer recommendations concerning the integration of video game technologies and programs, future research and practice, and knowledge dissemination.

**Conclusions:**

Digital game interventions employ unique, experiential, and interactive features that potentially improve skills and facilitate learning among players. Digital games may also provide a new treatment platform for youths with mental health conditions. Assessing current knowledge on video game technology and interventions may potentially improve the range of interventions offered by youth mental health services while supporting prevention, intervention, and treatment.

**International Registered Report Identifier (IRRID):**

PRR1-10.2196/13834

## Introduction

### Background

The risk of developing first-time mental health disorders is greatest among adolescents and young adults [1]. Early intervention provides a window of opportunity to promote detection, rapid access, monitoring, and treatment of youth mental health problems in the community [2-9]. Youth mental health programs designed to serve people between the ages of 11 and 25 years are rapidly emerging worldwide [6,9-13] and showing promising results in terms of improving mental health–related outcomes [5,14-16]. Several definitions of youth exist in the literature. For Statistics Canada and other international agencies, youth is considered to be a transition period in the life course between 16 and 29 years, involving a concentration of formative moments that occur in close succession (eg, achieving autonomy in relation to the family of origin, financial and residential autonomy, a stable couple relationship, family formation, and participation in society through full citizenship [17,18]). The literature on high-quality care in youth mental health programs calls for the following approaches: a community-based stepped care model of care; a youth-friendly physical space; stigma-free culture of care; and innovative information technology solutions to support the ability of programs to monitor the provision of high-quality care (measurement-based care) [6,9-13] and meet the needs of a generation that has grown up with digital technologies [19]. Youths in contact with mental health services strongly endorse the use of technology to receive information on medication, education, career, employment, and mental health, support their treatment journeys (eg, decision-making tools), and facilitate recovery [20].

Research in e-mental health is developing rapidly [21-23] despite slow uptake in clinical settings. Various reviews to date have identified how e-mental health tools may be effective in improving access to services, supporting evidence-based and personalized care, encouraging patient engagement, reducing stigma, and ensuring cost effectiveness [21-28]. These critical and comprehensive reviews have assessed various technologies, including telehealth, internet-based interventions or virtual communities, virtual reality and artificial intelligence, and video games or serious games [21-23,29]. What is needed, however, is a focused review that accurately assesses the treatment capacity, impact, and implementability of specific e-mental health solutions and technologies for youths. This review aims to address this need by exploring how digital game interventions may be integrated within current youth mental health services.

At any given moment, digital or video games are being played by millions of adolescents and young adults around the world. These games are among the preferred and most heavily used technologies among youths who access mental health services [30,31]. Not surprisingly, there is a growing body of literature that explores the advantages of playing digital games among players in different age groups, particularly in terms of improving attention span, memory, and problem-solving skills [32]; enhancing the ability of gamers to cope with failure [32] and manage emotions [32]; improving information retention [33]; facilitating deep learning [34]; supporting and promoting behavior change [35]; and promoting socialization [32]. Moreover, evidence suggests that games can be effectively used as therapeutic tools to support recovery from anxiety, depression, posttraumatic stress disorder (PTSD), and other disorders [36-42]. Finally, youths and primary care providers have identified video game technology as a valuable tool for early intervention and treatment [30,43].

Most studies have focused thus far on the negative impact of video games, describing potential harms related to aggression, addiction, and depression [44,45]. The World Health Organization, in the *International Classification of Diseases, 11th Revision*, recently identified a new classification of gaming disorder, defined as a pattern of gaming behavior (“digital-gaming” or “video-gaming”) characterized by impaired control over gaming, increasing priority given to gaming over other daily life activities, and continuation or escalation of gaming despite the occurrence of negative consequences. This new classification leaves health care providers with unique challenges related to assessment, diagnosis, and treatment for this disorder [46,47].

In light of these two contrasting realities—the desire of youths to play games and the evidence for gaming disorder—there is a need for critical assessment of the existing evidence on the use of digital game interventions within the context of mental health services for youths and the ways in which such interventions can be implemented in a safe and effective way.

### Objective

Systematic reviews and meta-analyses that assess the accessibility, feasibility, and effectiveness of serious video games for use in mental health have been published [36-42]. However, these reviews focused on specific mental health conditions, mainly depression and anxiety [36-42], without reporting on other mental illnesses affecting youths, such as psychosis, eating disorders, and substance misuse. Moreover, some of these reviews assessed the effectiveness of digital games for mental health in mixed-age populations, which often included older adults. Based on these gaps, the aim of the Gaming My Way to Recovery scoping review project is to identify and map the available evidence on the use of video game technologies for youths aged 11 to 29 years with mental health and substance use problems. This work complements and expands on existing systematic reviews by exploring the available evidence with respect to various mental health conditions affecting youths.

## Methods

### Objectives and Research Questions

This scoping review will use the stepped care model, a care delivery system in which treatment options are triaged based on relevant criteria (eg, severity) and shared decision-making practices [48,49], as a conceptual framework for mapping the available evidence on the use of digital game interventions. The stepped care model is often used in the youth mental health field to promote greater integration between mental health services, tailor treatments to youths, and ensure high-quality care.

In light of this model, our primary research question is as follows: For which youth mental health conditions have digital games been used and which broad objectives (eg, prevention, treatment) have they addressed?

Secondary research questions that will help generate a comprehensive picture of the available evidence are as follows: First, regarding equity, effectiveness, and impact: In which youth populations have digital game interventions been used and shown to be effective? What do we know about the experiences of youths and providers in receiving or providing mental health interventions through video game technologies? What are the current research gaps in this area? Second, regarding efficiency and process: How have digital game interventions been employed as prevention and treatment interventions in youth mental health services? What have been the outcomes of these programs? What barriers and enablers affect the use of digital or video games in clinical settings? Third, regarding engagement: What do we know about youth, family, and provider involvement in the creation and evaluation of digital game interventions for prevention and treatment of mental health conditions? Fourth, regarding relevance and sustainability: What evidence exists for the ability of digital game interventions to support youth mental health services? How can this evidence be used to support the integration of digital game interventions into youth mental health services? And fifth, regarding ethical practices: How may video game interventions be used in youth mental health services without doing harm?

### Scoping Review Method

To answer these research questions, we will conduct a scoping review that will systematically capture the main evidence, types of evidence, key concepts, models, and gaps in research on the use of digital game interventions for youths 11 to 29 years old with mental health and substance use problems. Scoping reviews are useful for the assessment of emerging evidence when it is still unclear whether more specific questions might be posed. A scoping review is the most suitable method for knowledge synthesis, as it considers different types of evidence and publications (from empirical studies, to reviews, to coverage of a complete body of literature) while also reporting on the types of evidence that inform practice in the field and the ways in which research has been conducted.

We will use the scoping review methodology developed by Arksey and O’Malley to guide this process [50]. Building on the Arksey and O’Malley framework, Levac and colleagues [51] further suggested that the consultation stage provide opportunities for stakeholder involvement, where insights beyond those reported in the literature may be provided. Therefore, we will engage partners and knowledge users, acting as consultants, throughout the scoping review.

#### Stepped Care as a Conceptual Framework

**Figure 1:**
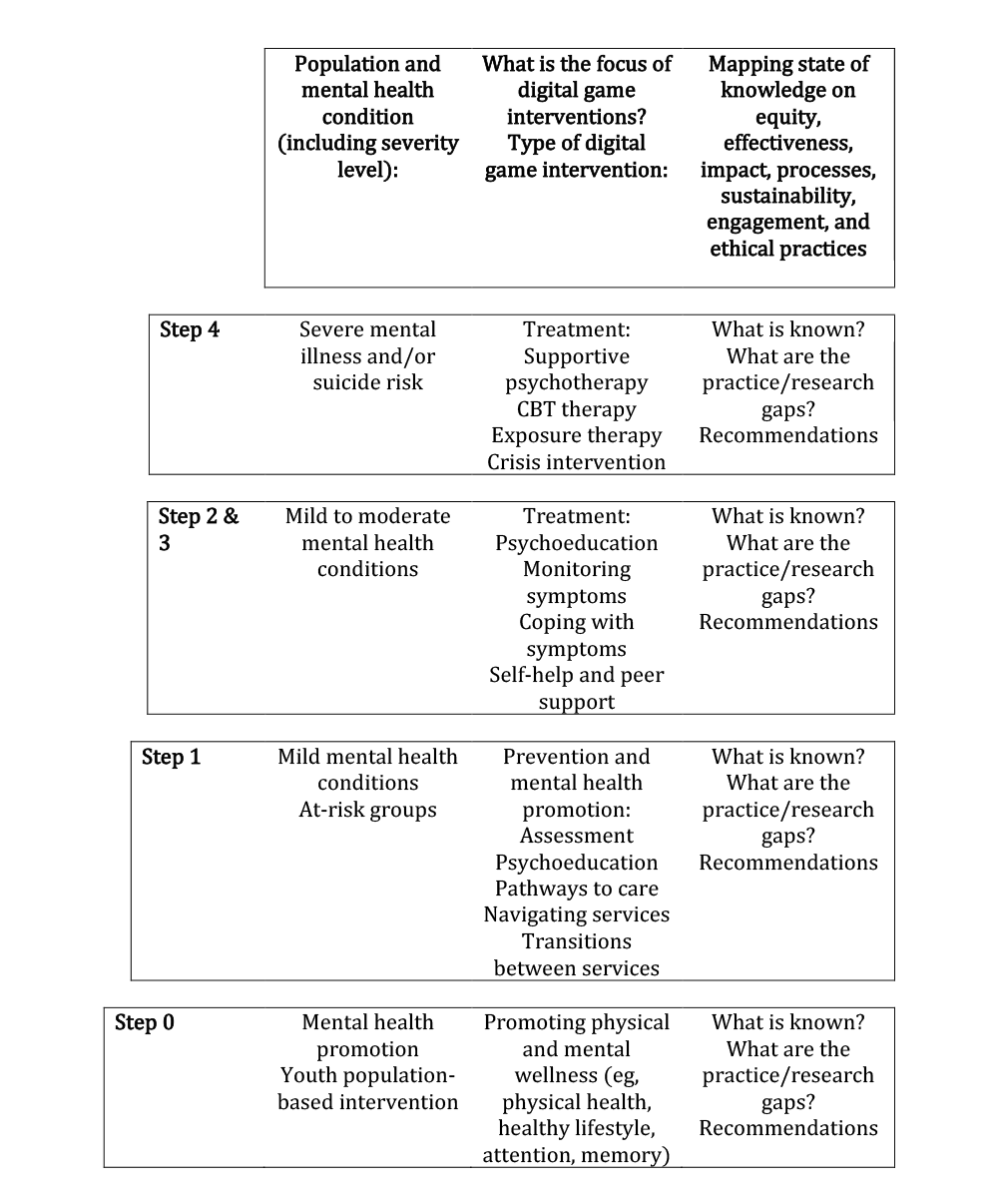
Model 1 adaptation of the traditional stepped care model. CBT: cognitive behavioral therapy.

This scoping review will use the stepped care model as a conceptual framework to systematically guide synthesis of the literature, identify existing evidence and gaps in knowledge, and provide recommendations for future studies and interventions. Model 1 (Figure 1) is our adaptation of the traditional stepped care model, which shows the targeted population and treatment options (from low-intensity to high-intensity treatment). This revised model brings together the aims and research questions posed in this knowledge synthesis project with the targeted population and treatment options. Using model 1, we will map the video game interventions that have been studied and the level of evidence for each video game intervention, looking at equity, effectiveness, impact, processes, sustainability, user engagement, and ethical practices, where possible.

#### Stakeholder Engagement

This knowledge synthesis project will engage stakeholders and partners (youths, family members or caregivers, and health care providers) within key stages of the project: proposal development, statement of aims and objectives, and data analysis and synthesis, as well as, importantly, the dissemination and translation of findings. The involvement of 3 different stakeholder groups will increase the relevance of the scoping review while enhancing its quality, innovation, and potential for implementation in youth mental health care settings. We will invite youths experiencing mental health problems who are also video game players to participate in this project and share their knowledge and expertise. They have unique experience and insight into their own health conditions and treatment, as well as knowledge and opinions on video game technologies (including associated ethical considerations). Caregivers (eg, family members and friends) are experts in how to care for and support those with mental illness or substance use problems and in knowledge of their needs. Thus, they may provide valuable perspectives on how video game interventions can help or hinder the recovery journey of affected family members and friends.

ACCESS Open Minds [16,52], a pan-Canadian network of 14 youth mental health service sites, will actively support this project. Youths and family members from the 3 ACCESS Open Minds Councils (National Youth Council, Family and Careers Council, and the Indigenous Council) and members of other youth networks will be invited to participate in the project. A project advisory group composed of 4 to 6 members (youths and family members interested in digital games as an intervention) will be established and meet 3 to 4 times over the course of the project. A peer researcher, a youth with lived experience and self-identified as a gamer, will help facilitate the meetings, which will cover the following topics: the nature of a scoping review, and a general discussion on project aims and how the knowledge of each advisory member may contribute to the project (first meeting); a review of data extraction forms and preliminary findings (second meeting); and the design of knowledge translation activities and materials, including contributions from the project advisory group (third and fourth meetings).

Stakeholders, especially youths, may provide insight on how to differentiate between playing video games for fun and the problems that may occur in relation to playing video games [53,54], suggesting which elements of the games should be further explored during the data extraction process. At the same time, stakeholders may benefit through their involvement in terms of their ability to access information and increase knowledge and skills concerning knowledge synthesis methodologies and processes.

Members of the project team are health care providers from 3 countries (Australia, Canada, and Denmark), who represent various youth mental health networks (ACCESS Open Minds, Youth Wellness Hubs Ontario, Frayme, Headspace, and Black Dog Institute). These youth networks will support dissemination of the project among their own members, as well as through professional associations such as the Early Psychosis Intervention Ontario Network and game research programs or groups (Participatory Research at McGill; Games and Gamification for Human Development & Well-being Working Group; and the Center for Computer Games Research at the IT University of Copenhagen).

#### Scoping Review Stages

The following section describes the 5 stages involved in the Arksey and O’Malley scoping review methodology.

##### Stage 1: Identifying the Research Question(s) and Revising the Protocol

We will present this scoping review protocol and proposed research questions to project partners and knowledge users, including youths, families, and providers, to gather feedback and identify missing elements. If required, we will revise the protocol to reflect the input of project partners and knowledge users during the different phases of the project.

##### Stage 2: Identifying Relevant Studies (Databases to be Searched and Strategies)

A systematic search strategy has been constructed by a health sciences librarian in consultation with the research team and reviewed by another librarian as recommended by the Peer Review of Electronic Search Strategies checklist [55]. This strategy will be run in MEDLINE (through Ovid) and adapted for CINAHL, EMBASE (Ovid), PsycINFO (Ovid), the Cochrane Library, and ProQuest Dissertations & Theses for all years from inception of each database to November 30, 2018. We will update the search of all relevant databases for the 12 months prior to publication of our results. No limits for language or publication type will be applied. We will not include gray literature in this project. We will use the citation software EndNote (Clarivate Analytics) to remove duplicate citations. Multimedia Appendix 1 presents the MEDLINE search strategy.

##### Stage 3: Study Selection

The review process will comprise 2 levels of screening: (1) a title and abstract review, and (2) a full-text review. We will use Rayyan (Qatar Computing Research institute) [56] to screen titles and abstracts. In the first phase, 2 research assistants will independently examine titles and abstracts for all retrieved citations according to the following inclusion criteria: (1) the intervention used a digital game delivered on any technical platform, including PCs, consoles (handheld console), mobile devices, and virtual reality; (2) the intervention targeted mental and substance use disorders, defined in terms of specific disorders, including mood disorders (eg, depression, bipolar disorders), anxiety disorders, obsessive-compulsive disorder, schizophrenia and related psychotic disorders, eating disorders, PTSD, and substance-related disorders; (3) the study pertained to an age group between 11 and 29 years [7,17,18,57]; (4) the study was published in English or French; and (5) the study was published between 2010 and 2020. Articles will be excluded if they (1) are theses or dissertations, (2) do not provide an abstract, (3) are conference presentations, (4) focus on a game intervention that targeted physical illnesses (eg, cancer, dementia, Alzheimer disease, epilepsy, and chronic pain), (5) primarily used telemedicine interventions, or (6) focused on commercial games for entertainment purposes only or were nondigital games (eg, board games).

We will retain studies identified as eligible for phase 2 (full-text review). In this phase, 2 raters will screen the full texts of articles to determine whether they meet the inclusion criteria listed above. We will retain eligible studies for data extraction in stage 4. Differences in ratings will be resolved through discussion until a consensus is reached. If agreement is not reached, the project team leader will intervene to make a final determination.

##### Stage 4: Data Collection and Extraction

A data extraction sheet will be developed by the research team and consulting partners by adapting relevant critical appraisal tools. This sheet will confirm the eligibility of each study and extract the relevant characteristics. Knowledge users will have the opportunity to provide input to confirm that the information for extraction is relevant. The variables to be extracted from the articles are divided into the following categories: (1) participant characteristics, including age, (2) study characteristics and methodology, (3) game characteristics, (4) youth and family engagement in the design of the digital game intervention, (5) youth and family engagement in the evaluation process, (6) treatment settings, (7) treatment level (health promotion; treatment for mild, moderate, and severe mental illness), (8) level of integration with in-person treatment, (9) game costs, and (10) sustainability. Specific participant and study characteristics include variables such as target group, recruitment, treatment type (single- or multicomponent interventions), primary outcome measures, type and extent of guidance provided during the intervention, setting of the intervention, study conditions, attrition, and results. Game characteristics will comprise variables such as the title of the game used in the study, serious game type, game genre, purpose of the game, and country where the study took place (country identification, and whether it is a low- to middle-income country or a high middle- to high-income country).

##### Stage 5: Data Summary and Synthesis of Results

This scoping review will map knowledge, evidence, and overall findings aligned with the proposed research questions. We will analyze each variable captured on the extraction form based on the type of data and using descriptive statistics, for example, percentages and thematic analysis. We will summarize the data using statistical plots and graphics, as well as through the creation of text tables, to describe key characteristics for each revised study (see Results section). We will further integrate the review findings into model 1. A revised model 1 will provide a synthesis of results for the available evidence on equity, effectiveness, impact, processes, efficiency, sustainability, engagement, and ethical practices in the use of digital game interventions for mental health and substance use conditions in youth populations.

#### Quality Assessment

Throughout the data extraction process, we will use well-known critical appraisal tools to ensure the extraction of essential information from the articles and to promote rigor. The critical appraisal tools that we plan to use, if appropriate, include Preferred Reporting Items for Systematic Reviews and Meta-Analyses for meta-analyses [58] and Grading of Recommendations, Assessment, Development and Evaluations II for guidelines [59]. When appropriate, for example in the case of randomized controlled trials, systematic reviews, and meta-analyses, we will use critical appraisal tools to assess the risk of bias and report our findings as part of the final report.

## Results

### Stage 1

We began the identification of relevant studies in November 2018. Stakeholder engagement began in December 2018. Data extraction (stage 1) was slowly carried out during the publication process for this protocol. We expect that the completion of stages 2 to 4 will take 1 to 2 months. After completion of stages 1 to 3, we will confirm the number of studies that meet the inclusion criteria (title and abstract); the number of studies that meet the inclusion criteria (full-text review); and the number of studies included in full-text data extraction. Stage 5 will include the summary and synthesis of the results using the conceptual framework (model 1) and will take approximately one month to complete.

### Anticipated Outcomes

The scoping review will provide knowledge on the use of digital game interventions for youths aged 11 to 29 years with mental health and substance use problems. More specifically, we will synthesize knowledge related to the following subthemes and subpopulations: (1) specific disorders (eg, anxiety, depression, psychosis, PTSD, eating disorders), (2) level of treatment (mental health promotion, prevention, treatment), (3) modality of treatment (self-help, psychoeducation, psychotherapy), (4) type of service (eg, mental health intervention, substance use treatment, primary care, employment or educational supports), (5) population (eg, indigenous; ethnoracial; lesbian, gay, bisexual, transsexual, transgender, intersexual, queer, questioning, 2-spirited; disabled, linguistic, low income), (6) settings (eg, community care, primary care, specialized services, rural or remote services), (7) ethical practices, and (8) level of user engagement.

We will revise and update this list of anticipated outcomes based on project findings.

In relation to specific knowledge mobilization outcomes, this review will (1) provide recommendations on how to best integrate video game technologies and programs into youth mental health settings and services, (2) identify research and practice gaps in the literature on video game technologies in youth mental health to inform future research projects, and (3) generate different knowledge dissemination materials to share project results with key knowledge users (eg, youths, caregivers, service providers), as well as pertinent partners, associations, and networks.

## Discussion

Overall, digital game interventions and solutions hold promise as learning machines [60] because of their ability to build on pedagogical principles (eg, experiential learning, active engagement of learners). They may employ unique features that facilitate learning processes, introduce new modalities to increase knowledge, and improve coping strategies and skills, while providing a more interactive and youth-friendly way to deliver treatments. This review on digital game interventions will provide evidence to assist mental health providers and policymakers in evaluating the potential risks of digital game interventions for youths experiencing mental health and substance use problems. Providers working in youth mental health settings may promote the implementation of digital game interventions in their services, if appropriate, to support prevention and treatment.

Methodological knowledge gained from this scoping review process will be useful in the systematic assessment of other e-mental health technologies: virtual communities, telemedicine, etc. Moreover, this scoping review project will gather valuable knowledge on how to involve different stakeholders in knowledge synthesis activities so that future reviews on e-technologies may use similar processes in seeking input from pertinent stakeholders.

Clinical knowledge gleaned from this review will also generate valuable knowledge on how, and for what purpose, video game interventions can be effectively implemented in youth services for mental health and substance misuse, as well as the appropriate conditions for doing so. The assessment of current knowledge on digital game interventions has the potential to improve treatment in youth mental health services.

## Appendix

Multimedia Appendix 1 MEDLINE search strategy.

Multimedia Appendix 2 Peer review report summary.

## Abbreviations

PTSD: posttraumatic stress disorder
