# Supplementary material for: Digital Game Interventions for Youth Mental Health Services (Gaming My Way to Recovery): Protocol for a Scoping Review
Source: JMIR Res Protoc. 2020 Jun 24;9(6):e13834. doi: 10.2196/13834 (PMC7381025; doi:10.2196/13834)
Supplement: Multimedia Appendix 1 [file resprot_v9i6e13834_app1.docx]

## Appendix 1

Ovid MEDLINE(R) and Epub Ahead of Print, In-Process & Other Non-Indexed Citations, Ovid MEDLINE(R) Daily <1946 to Present>

1 Mental Health/ (32049)

2 mental disorders/ (152778)

3 DEPRESSION/ (104484)

4 exp Depressive Disorder/ (100701)

5 anxiety disorders/ or obsessive-compulsive disorder/ (42955)

6 exp Schizophrenia/ (98606)

7 exp Psychotic Disorders/ (48590)

8 exp "Feeding and Eating Disorders"/ (28164)

9 Gambling/ (4904)

10 alcohol-related disorders/ or alcohol-induced disorders/ or alcoholism/ or binge drinking/ (78069)

11 exp substance-related disorders/ (261129)

12 Post-traumatic stress disorders/ (28824)

13 Mood Disorders/ (13278)

14 Bipolar disorder/ (37647)

15 (mental health or mental illness* or mentally ill or mental disorder* or depression or depressive or anxiety or problem drinking or alcoholism or binge drinking or schizophrenia or schizophrenic or psychosis or psychotic or obsessive compulsive or eating disorder* or bulimi* or anorexi* or gambling or post-traumatic stress or PTSD or substance abuse).tw,kf. (785701)

16 ((drinking or alcohol) adj2 (disorder or binge or binges or binging)).tw,kf. (9673)

17 or/1-16 (1149542)

18 Video Games/ (4416)

19 Virtual Reality/ (514)

20 (Video game* or videogame* or serious game* or virtual reality or gamification or gaming or game-based or Nintendo or wii or xbox or x box or Gameboy or game boy or playstation or virtual space*).tw,kf. (15689)

21 ((Therapeutic or online or internet or web or digital or computer* or phone or iphone or android or app or application or apps or applications or console or handheld or hand held) adj2 game*).tw,kf. (2388)

22 18 or 19 or 20 or 21 (18355)

23 17 and 22 (2505
