# Supplementary material for: Digital Game Interventions for Youth Mental Health Services (Gaming My Way to Recovery): Protocol for a Scoping Review
Source: JMIR Res Protoc. 2020 Jun 24;9(6):e13834. doi: 10.2196/13834 (PMC7381025; doi:10.2196/13834)
Supplement: Multimedia Appendix 2 [file resprot_v9i6e13834_app2.pdf]

FRAYME
CONNECT.
ENGAGE.
TRANSFORM.

1145 Carling Avenue
Ottawa, ON Canada K1Z 7K4
frayme.ca

September 4, 2018

Dr. Manuela Ferrari
Douglas Hospital Research Centre
6875 Boulevard Lasalle
Burland Pavillion
Verdun, Québec H4H 1R3

Dear Dr. Ferrari,

Your application entitled "Gaming my way to recovery: Understanding how to integrate video game technologies into youth mental health services", submitted to the *Request for Proposals: e-services within Integrated Youth Service Models* competition, has been considered by the NCE-IKTP Youth Mental Health (operating as "Frayme").

In July 2018, the Leadership Team reviewed and approved it to move to the KMb Committee, with a couple of slight revisions:

- Revise the proposal and research questions to create a more neutral approach/vision toward video game technology/intervention for youth mental health.
- Describe the potential of this project to inform and support stepped care activities currently underway by Frayme

The revised version of your proposal was received on August 2018. The KMb Committee reviewed the application. The KMb Committee, a Board subcommittee approved your proposal for Frayme support.

Sincerely,

Paula Robeson

Director of Operations
Frayme
